# Dendritic Cell Derived Cytokines in Human Natural Killer Cell Differentiation and Activation

**DOI:** 10.3389/fimmu.2013.00365

**Published:** 2013-11-11

**Authors:** Obinna Chijioke, Christian Münz

**Affiliations:** ^1^Viral Immunobiology, Institute of Experimental Immunology, University of Zurich, Zurich, Switzerland

**Keywords:** IL-18, IL-12, IL-15, IFN-alpha, immunological synapse, NKp30, DNAM-1

## Abstract

Dendritic cells (DCs) and natural killer (NK) cells shape each other’s functions early during immune responses. DCs activate NK cells and NK cells can mature or kill DCs. In this review we will discuss which DC and NK cell subsets are mainly affected by this interaction, where these encounters might take place and which signals are exchanged. Finally, we will point out what the clinical benefit of understanding this interaction might be and how it changed our view on NK cells as innate lymphocytes.

## Introduction

Natural killer (NK) cells have originally been described by their function to spontaneously lyse tumor and infected cells (1–3). However, it has recently become apparent that they require mostly cytokine mediated activation to differentiate into effector cells and execute different effector functions – at least in humans – dependent on their differentiation stage (4–6). Three prominent cell populations that trigger this NK cell activation and differentiation have been identified. These are dendritic cells (DCs), neutrophils, and CD4^+^ T cells (7–12). In this review we will focus on the interaction of NK cells with DCs, with an emphasis on its role in augmenting NK cell function during the innate phase of immune responses.

Human DCs are composed of different subpopulations (13) of which the two main subpopulations are conventional and plasmacytoid DCs (cDCs and pDCs). While pDCs are primarily found in primary and secondary lymphoid tissues, including bone marrow, thymus, lymph nodes, and spleen as well as blood in steady-state conditions, cDCs can be found both in lymphoid tissues and peripheral organs. Human cDCs can be subdivided in two additional subsets, CD1c^+^ (BDCA1^+^) and CD141^+^ (BDCA3^+^) DCs, which have now been found in the skin, liver, and lung, in addition to primary and secondary lymphoid tissues (14). In addition to these constitutive DC populations, which are at least to a substantial part dependent on Flt3L in their development (15, 16), inflammatory DCs can develop from monocytes. This DC lineage is dependent on GM-CSF for its development and therefore, GM-CSF constitutes an integral component of human monocyte-derived DC differentiation *in vitro* (16). Finally, Langerhans cells constitute a human DC population in the epidermis and at least in mice their steady-state maintenance is dependent on stromal IL-34 (17, 18). To fulfill their function DCs are equipped with molecules that sense the environment and in contrast to mice, the human DC populations have quite restricted expression patterns of pathogen associated molecular pattern (PAMP) receptors (19). For example, the toll-like receptor (TLR) nine for unmethylated DNA, which can be stimulated by CpG oligonucleotides, is only expressed by pDCs in humans, as is TLR7 for single-stranded RNA. In contrast, the double-stranded RNA receptor TLR3 is highest expressed on CD141^+^ cDCs and elicits high IL-12 and IFN-α/β/λ production from this subset. Interestingly, the IFN-α production by CD141^+^ cDCs reaches similar levels as IFN-α production from pDCs after TLR7 stimulation (20, 21). Therefore, all of these human DC populations need to be considered for NK cell activation and differentiation and will be discussed below.

## Human Natural Killer Cell Subset Distribution

Natural killer cell reactivity is guided by the balance of activating and inhibitory receptors (22). Both are acquired sequentially during development where inhibitory receptors are also instructive in NK cell education (23, 24). NK cell differentiation can in part be driven by both IL-15 and IL-2 in humans (25, 26). It is now assumed that the first functionally competent NK cell subset are CD56^bright^CD16^−^ NK cells, which have lost c-kit (CD117) and IL-7Rα (CD127) expression (26, 27). These seem to acquire the intermediate affinity activating FcγRIII/CD16, successively down-regulate the inhibitory HLA-E receptor NKG2A/CD94 and acquire more and more inhibitory killer immunoglobulin-like receptors (KIRs) upon differentiation (28). Interestingly, at any stage CD57 expression seems to terminally differentiate the respective NK cell subset and diminish its capacity to further expand. While CD56^bright^CD16^−^ NK cells respond to cytokine stimulation primarily with cytokine production, further differentiated CD56^dim^CD16^+^ NK cells display increased cytotoxicity and can produce a rapid, but transient cytokine burst upon tumor or infected cell encounter (25, 29). Interestingly, the successive up-regulation of KIRs seems to influence the reactivity of the later NK cell differentiation stages, depending on the expression of the cognate HLA class I ligands (24). Namely, NK cells with KIRs specific for self-HLA class I molecules have a higher reactivity against HLA class I negative tumor cell targets (30). These so-called licensed NK cells accumulate preferentially during some viral infection, primarily during persistent human cytomegalovirus (HCMV) infection (31). Some of these NK cell subset expansions allow for a more rapid response to secondary challenge with the same pathogen, which could be interpreted as an immunological memory function of the NK cell compartment (32, 33). These infection-experienced NK cells have been suggested to be enriched in the CXCR6 expressing hepatic NK cell subset, at least in mice (34).

The different NK cell differentiation stages have been found to be enriched at distinct anatomical sites (35). While CD56^dim^CD16^+^ NK cells predominate in the blood, most other tissues harbor high frequencies of CD56^bright^CD16^−^ NK cells. This IFN-γ producing NK cell subset has been originally found to mainly populate lymph nodes, tonsils, and splenic white pulp (25, 36–38). However, this NK cell differentiation stage has recently also been found to be enriched in liver, skin, uterus, joints, and tumor tissue (39–43). The CXCR6 positive NK cell subset with memory-like features might preferentially home to liver (34). Therefore, different NK cell differentiation stages can be preferentially found in distinct organs and their location might determine with which human DC populations they can preferentially interact.

## Sites of Interaction between Human DCs and NK Cells

While DCs can be found in all tissues, after activation, also called maturation, by for example TLR ligands, they migrate to or remain in secondary lymphoid tissues (13). Therefore, the interaction between mature DCs and resting NK cells would probably preferentially take place in secondary lymphoid tissues. Consistent with this notion, human NK cells and cDCs have been found to be enriched in the T cell zones of lymph nodes (36, 44). Moreover in mice, activation of NK cells in different infectious settings required DCs and homing of NK cells to secondary lymphoid tissues (45). Furthermore, injection of mature DCs resulted in the attraction of NK cells to secondary lymphoid tissues in mice (46) and brief contacts of NK cells with DCs have been observed in lymph nodes after adoptive transfer of mature DCs or *in vivo* activation with TLR3 and 4 ligands (47). Human secondary tissues might be especially predestined for these interactions, because CD56^bright^CD16^−^ NK cells preferentially home to these sites via CCR7 and CD62L expression and are enriched at these sites (36, 44). Moreover, mature monocyte-derived DCs preferentially stimulate CD56^bright^CD16^−^ NK cells to proliferate and produce cytokines (37, 44, 48). Thus, NK cell activation by mature DCs probably happens primarily in the T cell zones of secondary lymphoid tissues including lymph nodes.

Once activated, these NK cells might then leave secondary lymphoid tissues and home to sites of inflammation. Indeed, it has been observed that NK cells and DCs co-localize in inflamed skin (49). These activated NK cells might kill immature DCs at this site in order to prevent them from transmitting tolerogenic signals to secondary lymphoid tissues (50). Indeed, NK cell killing of preferentially immature DCs has been observed, especially when activated NK cells outnumber DCs (51, 52). In light of the fact that in most peripheral human tissues, inflamed organs, or tumor microenvironment, it has been shown that CD56^bright^CD16^−^KIR^−^ NK cells are enriched (35), it is interesting that this NK cell subset again might be preferentially killing DCs in the autologous setting without compromised MHC class I expression (53). However, NK cell reactivity might be curbed by regulatory T cells at these sites, who have been suggested to impair IL-2 and IL-15 mediated expansion and activation in mice (54–56). Furthermore, myeloid derived suppressor cells (MDSCs) might inhibit anti-tumor NK cell responses (57, 58) and their depletion by chemotherapeutika could augment their reactivity in tumors (57, 59). Only in conditions of infection induced down-regulation of MHC class I, as for example during HCMV infection, terminally differentiated KIR^+^NKG2C^+^CD57^+^ NK cells might accumulate and then be enriched in peripheral tissues (33). Thus, NK cells might be stimulated in secondary lymphoid tissues by mature DCs and afterward might kill immature DCs at peripheral sites. Since both immature and mature DCs express significant levels of MHC class I molecules as ligands for inhibitory NK cell receptors, like KIRs, preferentially CD56^bright^CD16^−^KIR^−^NK cells might be involved in both interactions.

## Signals in Human DC Interaction with NK Cells

**Figure 1:**
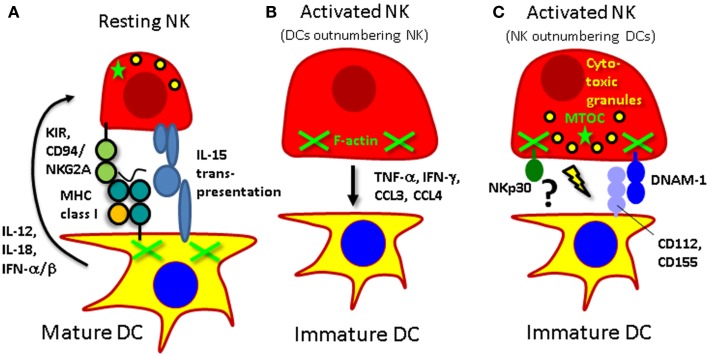
**Interactions of human NK cells with DCs**. **(A)** Mature DCs activate resting NK cells via IL-12, IL-15, and type I IFN. At the same time NK cells receive an inhibitory signal via killer immunoglobulin-like receptors (KIRs) or CD94/NKG2A to prevent them from killing mature DCs. **(B)** Activated NK cells can mature DCs via secretion of TNF-α, polarize them to produce IL-12 for Th1 induction with IFN-γ, and attract them via the CCR5 ligands CCL3 and CCL4. **(C)** If they, however, outnumber immature DCs they can kill these targets by perforin mediated lysis after engagement of the activating receptors NKp30 and DNAM-1.

According to these two different sites of interactions for DCs and NK cells, the stimulatory and killing signals that are exchanged require different molecules (Figure 1). In secondary lymphoid tissues, probably primarily cytokines are exchanged. Interleukin-12 and -18 have mainly been identified to activate cytokine production by NK cells (37, 44, 60). In response to IL-12, NK cells primarily produce IFN-γ, TNF-α, and GM-CSF. In contrast, IL-15 is involved in DC-stimulated NK cell proliferation, survival, and pre-activation (44, 45, 61). Interestingly, IL-15 only reaches the cell surface of the producing cell in complex with IL-15Rα (62), and trans-presentation might facilitate cell contact dependent IL-15 signaling. Finally, type I IFN augments NK cell cytotoxicity (37, 63). Depending on their cytokine secreting potential, different DC subsets are therefore capable of triggering one or the other NK cell function. PDCs stimulate primarily NK cell cytotoxicity via their type I IFN producing function (63). CD1c^+^ conventional and monocyte-derived human DCs are capable of producing IL-12, particularly after maturation with a TLR3 agonist (37, 63). Finally, Langerhans cells can support NK cell survival via their ability to present IL-15 on their surface (64). Thus, different human DC subsets stimulate distinct NK cell effector functions primarily via secretion of cytokines.

The respectively activated NK cells can then however signal back to DCs and presumably spread immune activation to neighboring secondary lymphoid tissue resident DCs (Figure 1). It has been shown that NK cell produced TNF-α can mature DCs (52, 65). This maturation can initiate adaptive T cell mediated immune responses against for example tumors (66, 67). Moreover, NK cell produced IFN-γ can assist in the polarization of Th1 responses by DCs (46, 68–70). Particularly, IL-18 activated NK cells up-regulate secondary lymphoid tissue homing markers like CCR7, and can stimulate IL-12 production by DCs (71). These NK cell stimulated DCs also up-regulate CCR7 and migrate in response to its ligand CCL21 (72). Furthermore, these so-called “helper” NK cells can also stimulate DCs to produce chemokines, primarily CXCL9, CXCL10, and CCL5, which allows attraction of effector CD8^+^ T cells (73). Therefore, NK cells can mature DCs to preferentially home to secondary lymphoid tissues and prime Th1 responses.

Finally, the third outcome of interactions between DCs and NK cells is killing of DCs (Figure 1). It has been shown that this occurs between activated NK cells and immature DCs (51–53). At least monocyte-derived DCs are recognized by activated NK cells via their NKp30 and DNAM-1 activating receptors (51, 74–76). Mature DCs are protected from this NK cell lysis by up-regulation of MHC class I molecules, including the non-classical HLA-E molecule (51, 53). Thus, DCs express ligands for activating receptors on human NK cells, but are after maturation protected from NK cell lysis by increased expression of MHC class I molecules.

## Immunological Synapses That Mediate NK Cell Interaction with DCs

Natural killer cells interact with target cells usually via the establishment of one of two types of immunological synapses. If activating signals dominate the interaction, an activating immunological synapse is observed with actin polymerization in the NK cell, polarization of the microtubule organizing center (MTOC) to the synapse and cytotoxic granule release through the center of the synapse, which leads to the killing of the target cell (77). On the contrary, if inhibitory signals prevail, inhibitory immunological synapses do not mature with cytoskeleton rearrangement, are short lived and the NK cell dissociates from the target cell without mobilizing any effector functions (78). NK cells also interact with DCs through immunological synapses (61, 79–81). However, the outcome of the interaction between mature DCs and NK cells is NK cell activation without killing of the conjugated DC. Therefore, we termed this immunological synapse regulatory. It seems to be designed to efficiently exchange paracrine IL-12, IL-18, and IL-15 from DCs to NK cells, in order to stimulate cytokine production and survival of NK cells (61, 79, 80). This becomes especially important when maturation stimuli allow DCs only to produce limited amounts of these cytokines and other leukocyte populations in the lymph node environment can consume these cytokines in addition to NK cells (37, 79). At the same time, inhibitory interactions are exchanged at the regulatory immunological synapse between mature DCs and NK cells. Inhibitory receptors like KIRs accumulate in other membrane domains than NK cell stimulatory IL-15/IL-15Rα complexes, although both are located in the center of immunological synapses of mature DCs with resting NK cells (61). This compartmentalization of inhibitory and activating domains occurs rapidly within 5 min after interaction between these two leukocyte populations. Upon longer interaction, the immunological synapse between mature DCs and NK cells is then stabilized by cytoskeletal rearrangements, including actin polymerization at the synapse in the conjugated DC (81). Interestingly, these cytoskeletal rearrangements seem to primarily support the inhibitory signals that are exchanged at the synapse between DCs and NK cells, because inhibition of actin polymerization in DCs by for example decreasing the expression of Wiskott Aldrich Syndrome Protein (WASP), which organizes the actin cytoskeleton at immunological synapses, leads to conversion of the immunological synapse into an activating NK cell synapse with actin polymerization in the conjugated NK cells and killing of DCs. Therefore, human DCs seem to coordinate their interaction with NK cells via a regulatory immunological synapse, which allows exchanging at the same time stimulatory signals for NK cells and signals that inhibit them from killing DCs.

While these long-lasting synapses have been observed with human cells *in vitro*, DCs, and NK cells establish only short interactions, usually below 3 min, in mouse lymph nodes (47). It is so far unknown, which species differences might cause these divergent interaction kinetics. One possibility, however, could be that the possible NK cell subpopulation counterpart in mice (82) of the CD56^bright^CD16^−^ NK cell population, which preferentially forms conjugates with human mature DCs (61), engages in these long-lasting synapses, and the respective murine NK cell subset is too rare to be readily observed in mouse lymph nodes *in vivo*. Alternatively, however, the short interactions *in vivo* could also result from additional stimuli like chemokine gradients that could sustain NK cell mobility and shorten NK cell interactions with DCs. Further *in vivo* imaging studies could clarify such heterogeneity of immunological synapse formation between DC and NK cell subpopulations.

## Therapeutic Potential of NK Cell Interactions with DCs

Both NK cell activating as well as DC restricting functions in the interaction of NK cells with DCs might be harnessed for therapeutic benefit. NK cell activation by DCs during vaccination might generate a stimulatory environment for the priming of Th1 responses. Along these lines, TLR3 agonists mature DCs for optimal NK cell stimulation *in vitro* (37). Moreover, synthetic double-stranded RNA induced a profile beneficial for NK cell stimulation in healthy volunteers (83) and was able to augment NK cell responses against tumor cells in mice with reconstituted human immune system components (84). Therefore, the right choice of adjuvant could harness NK cells during vaccination. Cytokine production by activated NK cells can improve maturation of DCs to expand tumor specific T cells more efficiently and acquire homing markers for secondary lymphoid tissues (72). Thus, NK cell activation by DCs during vaccination could feed-back to antigen presenting cells to increase their Th1 polarizing potential.

However, a completely different clinical benefit of DC interaction with NK cells was revealed when it was noticed that alloreactive NK cell therapy by haploidentical bone marrow transplantation against acute myeloid leukemia (AML) relapse also diminished graft-versus-host-disease (GvHD) (85, 86). It was noted that NK cells were not only able to target HLA mismatched leukemia cells, but also allogeneic DCs, which then no longer can prime donor T cells, specific for the host MHC allotype, to attack the host. This NK cell reactivity against MHC mismatched DCs might also be beneficial in other transplantation settings. At least in experimental animal models, it is well documented that alloreactive NK cells eliminate DCs from allogeneic grafts (87–90). In MHC mismatched skin, pancreatic β-islet and lung transplantation, it was shown that host NK cells eliminate donor DCs from the transplant, which subsequently led to decreased priming of host derived alloreactive T cell responses. The resulting diminished rejection allowed the respective transplants to survive longer and to perform better. These data suggest that allogeneic DC targeting by NK cells that lack KIRs against the MHC haplotype of the graft can ameliorate GvHD by donor NK cell cytotoxicity or transplant rejection by host NK cell cytotoxicity. These clinical benefits might be augmented by adoptively transferring alloreactive NK cell lines, which could be either stimulated with TLR3 agonist matured DC populations or their cytokines *in vitro*. IL-12, IL-15, IL-18, and IFN-α should be considered as stimulatory monokines that could be used to expand functionally competent NK cell lines *in vitro*. Adoptively transferred NK cell lines that have been activated and expanded in this fashion might confer protection against leukemia relapse and GvHD in a haploidentical transplantation setting until NK cell populations have reconstituted from transplanted hematopoietic progenitor cells.

## Conclusion

In recent years it has become apparent that DCs can in addition to being superior antigen presenting cells for T cell priming, activate innate lymphocytes (69). In fact, the parallels between CD8^+^ T cell priming and NK cell activation by DCs are quite striking. For both lymphocyte populations, activation happens in secondary lymphoid tissues, is dependent on IL-12 and requires IL-15 for survival (91). DCs form immunological synapses with CD8^+^ T cells and NK cells, which are stabilized by the DC cytoskeleton. Furthermore, both of them acquire cytotoxicity through this activation and loose initial cytokine production during further differentiation. Alongside, and presumably as a protective mechanism against immunopathology mediated by these cytotoxic lymphocytes, both CD8^+^ T cells and NK cells up-regulate inhibitory receptors, which safe-guard their activation upon target cell encounter. Finally, they both can kill DCs either after viral antigen presentation or virus induced MHC class I down-regulation. Therefore, it is tempting to speculate that NK cells are the evolutionarily older cousins of CD8^+^ T cells. However, it still needs to be clarified if they can also develop some sort of memory to infections through for example NK cell subset expansion in response to pathogens (33).

## Conflict of Interest Statement

The authors declare that the research was conducted in the absence of any commercial or financial relationships that could be construed as a potential conflict of interest.
